# Sporotrichosis Presenting as a Severe Ulcer in an Elderly Diabetic Man

**DOI:** 10.4269/ajtmh.24-0417

**Published:** 2024-10-29

**Authors:** Xiujiao Xia, Zehu Liu, Hong Shen

**Affiliations:** Department of Dermatology, Hangzhou Third People’s Hospital, Hangzhou Third Hospital Affiliated to Zhejiang Chinese Medical University, Hangzhou, China

**Figure 1.**
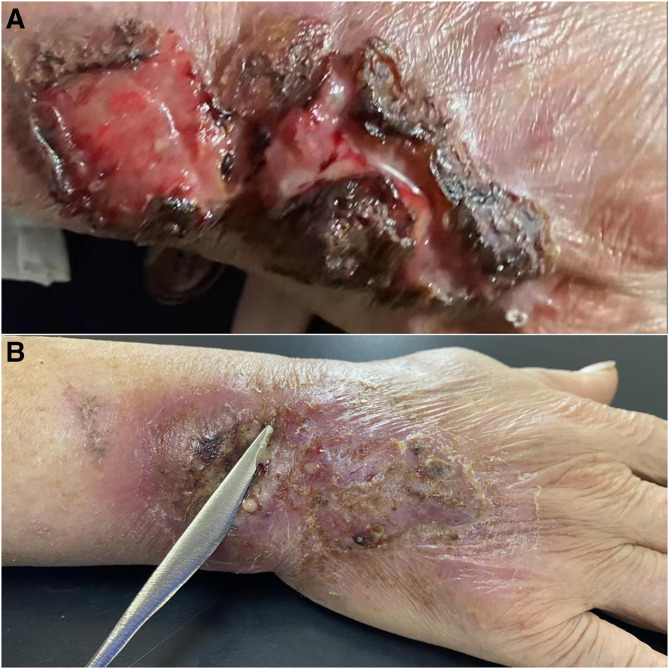
(**A**) Initial appearance of the lesion during the first visit to a local clinic, showing a marked ulcer (provided by the patient). (**B**) Appearance of the lesion after 6 months of treatment at a local clinic, showing an erythematous plaque with purulent discharge upon squeezing.

**Figure 2.**
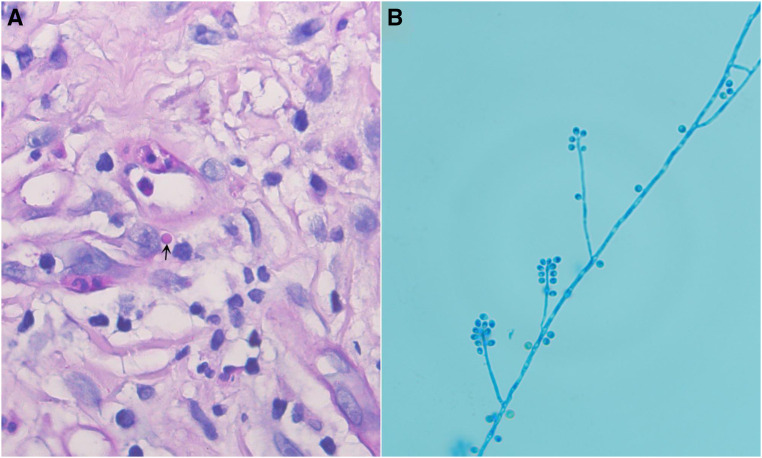
(**A**) Histopathology showing round yeast (arrow) within the cytoplasm of a giant cell (PAS ×1,000). (**B**) Slide culture of *Sporothrix globosa* on potato dextrose agar at 25°C on day 7, stained with lactic acid cotton blue (×1,000). PAS = periodic acid-Schiff.

An 85-year-old man with a 30-year history of type 2 diabetes mellitus, residing in a rural area, presented with a painful ulcerative lesion on his right hand that had persisted for 1 year (Figure 1A). He denied any trauma to his hand and had previously been evaluated at a local clinic, where he was treated with topical butenafine and polysulfonic mucopolysaccharide ointment, as well as oral minocycline hydrochloride (100 mg/day) for suspected pyoderma gangrenosum (PG). The lesion slightly improved after 6 months of treatment. Subsequently, the patient was referred to the Dermatology Department of our hospital. Examination of his right hand revealed a raised, approximately 10 × 4–cm, ulcerated lesion with pus discharge (Figure 1B). Routine hematological and biochemical investigations showed a C-reactive protein level of 18.8 mg/L (*N* <10 mg/L), random blood glucose of 19.71 mmol/L (3.9–11.1 mmol/L), alanine aminotransferase of 130 U/L (9–50 U/L), and aspartate aminotransferase of 157 U/L (15–40 U/L). Investigations for systemic diseases, including inflammatory bowel disease, were negative. Histopathology identified round, periodic acid-Schiff–positive yeast within the cytoplasm of a giant cell (Figure 2A). Tissue and pus culture on Sabouraud dextrose agar at 25°C for 10 days grew colonies of *Sporothrix globosa* (Figure 2B; GenBank accession number PP188558). Finally, a diagnosis of fixed cutaneous sporotrichosis was confirmed. Owing to the elevated aminotransferase levels, after thorough communication with the patient, we decided to administer 10% potassium iodide treatment at a dose of 30 mL/day. The ulcer completely resolved after 6 months of treatment.

Sporotrichosis is a subacute or chronic fungal infection caused by the dimorphic fungi of the genus *Sporothrix*. The clinical manifestations of sporotrichosis may vary depending on the immunological status of the host, the load and depth of the inoculum, the pathogenicity, and the thermal tolerance of the strain, among other factors.^1^ Diabetic patients are more susceptible to severe infections as a result of changes in skin trophism and impaired specific and nonspecific local defenses.^2^ Sporotrichosis can present with severe cutaneous ulcerations, mimicking noninfectious skin conditions such as PG,^3^^–^^5^ as demonstrated in this case. Pyoderma gangrenosum is a rare neutrophilic skin disease characterized by a painful ulcerative skin disorder.^5^ In this patient, the ulcerative and painful nature of the lesion led to a misdiagnosis of PG. This case highlights the necessity of considering cutaneous sporotrichosis in the differential diagnosis of cutaneous ulcers and the importance of biopsy and tissue culture, particularly if the condition is unresponsive to first-line therapies.
